# Cardiology and COVID-19: do we have sufficient information?

**DOI:** 10.2217/fca-2020-0126

**Published:** 2020-10-30

**Authors:** Nikita Garg, Brendan McClafferty, Devyani Ramgobin, Reshma Golamari, Rahul Jain, Rohit Jain

**Affiliations:** ^1^Lady Hardinge Medical College, New Delhi, Delhi 110001, India; ^2^Lake Erie College of Osteopathic Medicine, Erie, PA 16509, USA; ^3^Touro College of Osteopathic Medicine, New York, NY 10006, USA; ^4^Department of Hospital Medicine, Penn State Milton S. Hershey Medical Center, Hershey, PA 17033, USA; ^5^Department of Cardiology Indiana University, Bloomington, IN 47405, USA

**Keywords:** ACE-2, cardiology, COVID-19, SARS-CoV-2

## Abstract

Novel coronavirus disease 2019 (COVID-19) caused by severe acute respiratory syndrome coronavirus 2, which originated in Wuhan (China), transformed into a worldwide pandemic. The short span associated with the spread of the virus and its varied manifestations presents a steep learning curve for many clinicians on the front-line of treatment. Cardiology is one such affected area. This paper details the signs and symptoms of cardiovascular disease resulting from COVID-19, including its proposed pathophysiology, signs and symptoms, treatments and outcomes under investigation. The consensus is that COVID-19 patients with cardiovascular injury have a shorter duration from symptom onset to deterioration, higher mortality and higher prevalence in older populations. Diagnosis and intervention for patients with underlying cardiovascular comorbidities is critical.

Coronavirus disease 2019 (COVID-19) caused by severe acute respiratory syndrome coronavirus 2 (SARS-CoV-2) was originally recognized as a disease that affects the lungs and may cause complications, such as acute respiratory distress syndrome [1,2]. Initially, this disease was believed to present with fever and a cough potentially progressing to pneumonia. However, multiple presentations involving a myriad of systems have now been recognized as the disease spreads to over 36 million cases and has contributed to over 1,054,000 deaths as of 7 October 2020 [3]. COVID-19 interacts and affects cardiovascular systems at various levels via the angiotensin-converting enzyme 2 (ACE-2) receptor gaining entry to the host cells, causing myocardial injury and cardiac dysfunction [4]. ACE-2 is expressed mainly in the endothelium of the lungs, kidney and heart, which explains the high incidence of cardiac dysfunction [5].

Cardiovascular damage can also occur without signs and symptoms of respiratory infection [6]. The frequency and intensity of the respiratory complaints about COVID-19 patients is worse in patients with pre-existing cardiovascular issues and can even cause new-onset cardiac dysfunction [7–9]. This article aims to demonstrate a contemporary understanding of proposed pathophysiology, signs and symptoms, outcomes and treatment of patients with cardiovascular injury due to COVID-19.

## Proposed pathophysiology

There are multiple ways in which cardiovascular injury can occur due to this virus-

- Direct myocardial injury: Murine models affected with SARS viruses have shown that ACE-2 dependent myocardial infection occurs leading to decreased ACE-2 expression [10]. Macrophage infiltration also occurs in the heart leading to direct myocardial damage [11]. The direct damage can lead to myocarditis as demonstrated by increased wall thickness, diffuse biventricular hypokinesis and left ventricular dysfunction on cardiac MRI. ECG findings demonstrated diffuse ST-elevation in various studies. Laboratory analysis also has demonstrated elevated high-sensitivity troponin T and NT-proBNP [6,11].
- Indirect myocardial injury: This may occur via multiple mechanisms including direct damage to the myocardium in the setting of cytokine release related to activation of Type 1 and Type 2 T-helper cells. Patients also have higher levels of C-reactive protein (CRP), procalcitonin, ferritin, D-dimer, IL-2, IL-7, granulocyte-colony stimulating factor, IgG-induced protein 10, chemokine ligand 3 and TNF-α [12–14]. Additional cardiovascular considerations for these patients are hypoxemia secondary to respiratory failure, microvascular thrombosis due to hypercoagulability, adverse effects of various antiviral or corticosteroid medications, rupture of plaque and myocardial infarction via systemic inflammation, stress cardiomyopathy, electrolyte imbalance and hemodynamic derangement [9,15,16]. Comprehending the full scope of indirect injury to the myocardium because of SARS-CoV-2 infection will require further investigation into the interplay of how the various cytokines, proteins, systemic inflammation, etc. interact during and after an acute illness from SARS-CoV-2.

## Risk factors

Underlying cardiac disease is one of the major risk factors in acquiring COVID-19. One of the earlier studies done in Wuhan showed that pre-existing cardiovascular disease had an associated case fatality rate of 10.5% [17]. In a study done by Shi *et al.* looking at cardiac injury and mortality, 10.6% of the population had underlying coronary heart disease and 4.1% had heart failure [12]. Underlying cardiac disease is also associated with a higher prevalence of infection, accounting for up to ∼42% in another US-based study by Arentz *et al.* [18]. A separate study indicated that cardiocerebrovascular disease accounted for 40% of populations affected by COVID-19 as evidenced by Chen *et al.* [19]. Specifically, pre-existing disease processes, such as acute coronary syndrome, myocardial infarction, myocarditis, reverse Takotsubo syndrome, left ventricular systolic dysfunction, heart failure and arrhythmias, are also reported to be exacerbated by COVID-19 virus [12,20,21].

## Signs & symptoms of cardiovascular injury

The signs and symptoms of cardiovascular injury from this disease range from chest pain, dyspnea, palpitations, hypotension, cardiogenic shock, to sudden cardiac death [22,23]. Patients presenting with symptoms such as dyspnea and hypoxia without evidence of infection may be incorrectly presumed as having alternate respiratory sources of illness when in fact, they may have a concurrent cardiac etiology. These patients must have troponin and NT-pro-BNP along with other inflammatory biomarkers evaluated.

When it comes to signs, acute cardiac injury is defined as a high-sensitivity troponin I (TnI) above the 99th percentile upper reference limit is the most common [9]. Guo *et al.* studied 187 COVID-19 patients, of whom 52 (27.8%) had a myocardial injury as determined by elevated levels of TnT [24]. The incidence of cardiac injury ranged anywhere between 8 and 12% in various studies; the incidence being 13-fold higher in the ICU/severe category [8,25]. Moreover, the patients admitted to the ICU had a 2.2-fold higher troponin level when compared with the nonintensive care units. Also, patients who neared death had significantly elevated TnT and NT-proBNP levels when compared with admission labs [24].

Cardiomyopathy developed in around 33% of patients in a study by Arentz *et al.* [18]. Dong *et al.* reported four cases of heart failure. Two cases presented with severe symptoms and two with mild symptoms. The patients who were critically ill had a troponin I level of more than 20-fold [21]. Cardiogenic shock accounted for 7% fatalities in a study by Ruan *et al.* [26]. There was also a report of a case of fulminant myocarditis as evidenced by myocardial wall edema and extensive transmural late gadolinium enhancement on the chest MRI [27].

COVID-19 patients have an increased risk of ST-segment elevation myocardial infarction (STEMI) with variable presentations and were noted to have a higher prevalence of nonobstructive disease and a poor prognosis [28].

Temporary occurrence of S1Q3T3 and subsequent transient, nearly complete A-V block may reflect transient pulmonary artery hypertension secondary to trachea secretive obstruction, which may cause extensive small pulmonary artery compression [29]. The development of ST-elevation and ventricular tachycardia can have multiple triggers, such as hypoxia, hydroxychloroquine-induced arrhythmias, among others [29]. A study carried out by Wang *et al.* reported a 16.7% incidence of arrhythmia, more so in the intensive care unit (ICU) admissions [1].

High prevalence of thrombosis (16.7%) has been noted in a prospective cohort study, particularly in COVID-19 patients admitted to the ICU for hypoxemic acute respiratory failure even despite theraputic or prophylactic anticoagulation. In addition, the incidence of pulmonary embolism was higher in COVID-19 acute respiratory distress syndrome patients (11.7%) compared with non-COVID-19 patients (2.1%). This study discussed how the mechanism involved in COVID-19 thrombosis remains unclear despite obvious endothelial inflammation as evidenced by high leves of von Wilibrand factor antigen and factor VIII. They hypothesized that hypoxemia in pulmonary capillaries leads to vasoconstriction reducing blood flow promoting occlusion in addition to activation of hypoxia-inducible factors inhibiting tissue factor and plasminogen-activator inhibitor-1 [30].

## Treatment

For many patients with COVID-19, treatment has been mainly supportive and directed at relieving the associated symptoms of the virus. This holds for patients with mild clinical presentations who can manage themselves at home. For those who develop more severe complications such as acute respiratory distress syndrome (ARDS), sepsis, pneumonia and acute kidney injury, hospitalization is indicated [31].

In terms of cardiac injury, prompt evaluation with cardiac biomarkers (as discussed above), echocardiogram and or a coronary angiogram should be made depending on patient presentation. Earlier in 2020, the controversy surrounding the continued use of ACE inhibitors/angiotensin receptor blockers existed; however, the current consensus is that these medications should be continued in older patients with increased risk of heart failure or patients with frank heart failure [32,33]. Also, a large cohort analysis of 5894 SARS-CoV-2 positive patients found no substantial increase in the likelihood of a positive COVID-19 test or risk of severe COVID-19 associated with five common classes of antihypertensive medications [34].

Remdesivir has been shown to have activity against SARS-CoV-2* in vitro* [35]. Preliminary results of a randomized placebo-controlled trial for Remdesivir involving 1063 patients by the US National Institute of Allergy and Infectious Diseases indicated that treatment with this medication resulted in a 31% faster time to recovery (median of 11 vs 15 days with placebo) for patients to be discharged and no longer require supplemental oxygen (p < 0.001) [36]. However, there was also a double-blind randomized study of 237 patients with severe COVID-19 that indicated that Remdesivir compared with placebo for 10 days did not show a statistically different time to clinical improvement [37]. This same study indicated that a higher proportion of patients being treated with Remdesivir than placebo had dosing stopped prematurely because of adverse events such as aminotransferase or bilirubin increases, anorexia, nausea, vomiting and worsened cardiopulmonary status. We expect the further investigation into this medication to provide deeper insight as to its role in treating COVID-19 patients with cardiopulmonary disease.

In patients who have severe disease progression, vasopressors, mechanical ventilation and extracorporeal membrane oxygenation (ECMO) have proven to be successful [38].

Clinical trials are also underway to further investigate the role of convalescent plasma therapy (CPT). A systematic review of 5 studies encompassing 27 patients reported that all studies found CPT significantly reduced the viral load and increased the level of neutralizing antibodies over time. Also, all studies reported zero mortality after receiving CPT at varying doses. However, the authors of the review indicated that while the studies reported good outcomes, they were considered at risk of bias due to a combination of non-randomized evaluations, duration of therapy, dosage, confounding and poor methodological conduct for participant selection [39].

A recent study of 2104 patients who received dexamethasone and 4321 patients receiving usual care demonstrated a one-third reduced 28-day mortality in patients who received invasive mechanical ventilation (29.0 vs 40.7%, RR 0.65 [95% CI: 0.51–0.82]; p < 0.001). This is compared with one-fifth reduced 28-day mortality in patients who received oxygen without invasive mechanical ventilation (21.5 vs 25.0%, RR 0.80 [95% CI: 0.70–0.92]; p = 0.002). No change in mortality was demonstrated in patients that did not receive respiratory support (17.0 vs 13.2%, RR 1.22 [95% CI: 0.93–1.61]; p = 0.14) [40].

Regarding diagnosis and treatment of venous thromboembolism in COVID-19 patients, an article published by the *Journal of Thrombosis and Haemostasias* in May of 2020 recommended following standard-of-care objective testing for diagnosis of suspected venous thromboembolism based on clinical index of suspicion in COVID-19 patients [41]. Venous thromboembolism prophylaxis in nonintensive care unit hospitalized COVID-19 patients is recommended (Table 1). For these patients, routine thromboprophylaxis with low-molecular weight heparin or standard dose unfractionated heparin was the treatment of choice. For patients that are in the intensive care unit, the study recommended prophylactic-dose unfractionated heparin or low-molecular weight heparin. High risk bleed patients were recommended to receive intermediate-dose low-molecular weight heparin. The study indicated that treatment dose heparin should not be considered until further trials are available in this setting. Mechanical thromboprophylaxis (intermittent pneumonic compression devices) was also recommended. Duration of venous thromboembolism for hospitalized COVID-19 patients was recommended for all hospitalized COVID-19 patients that meet high venous thromboembolism risk criteria with duration of post-discharge prophylaxis of approximately 14–30 days [41].

**Table 1.** Recommended Venous Thromboembolism Prophylaxis for COVID-19 Patients After Assessment of Bleed Risk.

Non-ICU, hospitalized COVID-19 patient	ICU, hospitalized COVID-19 patients
Routine thromboprophylaxis with low-molecular-weight heparin or standard dose unfractionated heparin	Prophylactic-dose unfractionated heparin or low-molecular-weight heparin. High-risk bleed patients can receive intermediate-dose low-molecular-weight heparin. Treatment dose heparin should NOT be considered until further trials are available. Mechanical thromboprophylaxis (intermittent pneumonic compression devices) are to be considered

Prophylaxis modifications should be considered on extremes of body weight, deteriorating renal function or severe thrombocytopenia.

COVID-19: Coronavirus disease 2019; ICU: Intensive care unit.

## Outcomes

A study by Guo *et al.* demonstrated that patients with elevated troponin T (TnT) levels and underlying cardiovascular disease had the highest case fatality (25 of 36 [69.4%]) compared with (6 of 16 [37.5%]) in those with elevated troponin but without the pre-existing disease. Mortality was significantly higher in those with elevated TnT levels (59.6%, i.e., 31 of 52) than in those with normal TnT levels (8.9%, i.e., 12 of 135) [24]. It should be noted that patients with cardiovascular diseases have a significantly increased risk of death when infected with SARS-CoV-2 (p < 0.001) [26].

**Figure 1.**
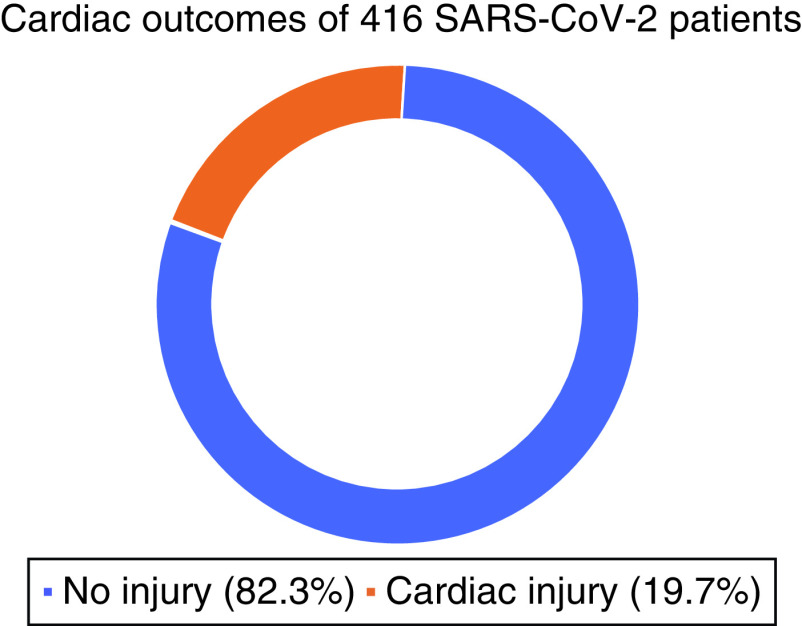
Percent of patients who suffered a cardiac injury according to cohort study by Shi *et al.* With cardiac injury determined via patients displaying higher inflammatory markers and other cardiac biomarkers, such as CRP, procalcitonin, CK-MB, myohemoglobin, TnI and NT-proBNP. SARS-CoV-2: Severe acute respiratory syndrome coronavirus 2. Created using data from [12].

In a cohort study by Shi *et al.* specifically looking at outcomes, 19.7% of patients had a cardiac injury (Figure 1). The patients with cardiac injury had higher inflammatory markers and other cardiac biomarkers, such as CRP, procalcitonin, CK-MB, myohemoglobin (MYO), TnI and NT-proBNP. These patients required greater proportions of invasive and noninvasive mechanical ventilation when compared with patients without cardiac injury. The same study also noted a higher risk of death at the time of symptom onset, as well as higher admissions in patients with underlying cardiac dysfunction [12].

Similarly, an analysis of heart injury laboratory parameters in 273 COVID-19 patients, researchers found that elevated concentrations of CK-MB, MYO, Tnl and NT-proBNP in venous blood were associated with the severity and case fatality rate in COVID-19 [42].

In a risk-adjusted study, NT-pro-BNP was found to be independently correlated with in-hospital death [43].

## Conclusion

Cardiac dysfunction is a well-known complication of COVID-19. ACE-2 receptors present in the heart may be the source of viral entry leading to myocardial damage. Pre-existing cardiac diseases in any form may predispose patients to be more susceptible to acquiring SARS-CoV-2. Cardiac injury can be measured by cardiac biomarkers such as troponins and NT-Pro-BNP and the presence of elevated cardiac biomarkers may indicate a worse prognosis. These cardiac biomarker elevations may be correlated with a rise in other inflammatory makers. The interplay between these cardiac biomarkers and inflammatory markers is under investigation. With the proposed mechanisms of myocardial injury summarized in Box 1. Clinical trials to evaluate multiple therapies are underway and their role in cardiology will become more apparent as research progresses. Management at this time is mainly supportive, in severe cases, ECMO may be utilized.

Box 1. **Proposed pathophysiology of cardiovascular injury.**

**Direct myocardial injury**

1. Macrophage infiltration resulting in myocarditis (increased wall thickness, diffuse biventricular hypokinesis and left ventricular dysfunction)

**Indirect myocardial injury**

1. Damage resulting from cytokine release related to T-cell activation
2.  Hypoxemia secondary to respiratory failure
3. Microvascular thrombosis
4. Adverse effects of medications
5. Plaque rupture
6. Myocardial infarction
7. Stress cardiomyopathy
8. Electrolyte imbalances
9. Hemodynamic derangement

## Future perspective

We believe that over the course of the next 5–10 years, the work of scientists around the world will continue to illuminate the exact pathophysiology behind the various cardiovascular sequela of COVID-19. As understanding of the disease improves, recommendations for treatment will also continue to evolve. Potential negative impacts of these therapeutic interventions on the cardiovascular system may not present for years. In addition, new therapies will continue to arise with currently unknown benefits to prevention and treatment of cardiovascular complications of COVID-19. Finally, we expect larger retrospective studies to shed light into what worked to protect patients and what may have caused increased mortality.

**Executive summary**

**Background**

- Coronavirus disease 2019 (COVID-19) caused by severe acute respiratory syndrome coronavirus 2 (SARS-CoV-2) was originally recognized as a disease that affects the lungs and may cause complications such as acute respiratory distress syndrome.
- Cardiology is an area of medicine that has been greatly impacted by this disease.

**Proposed pathophysiology**

- Direct damage via macrophage infiltration resulting in myocarditis (increased wall thickness, diffuse biventricular hypokinesis and left ventricular dysfunction).
- Indirect damage via cytokine release related to T-cell activation, hypoxemia secondary to respiratory failure, microvascular thrombosis, adverse effects of medications, plaque rupture, myocardial infarction, stress cardiomyopathy, electrolyte imbalances and hemodynamic derangement.

**Risk factors**

- Underlying cardiovascular disease is associated with increased mortality, as well as a higher prevalence of infection.

**Signs & symptoms**

- Cardiovascular symptoms from COVID-19 range from chest pain, dyspnea, palpitations, hypotension, cardiogenic shock, to sudden cardiac death.

**Treatment**

- Preliminary results of treatments showing improved patient outcomes at this time include supportive therapy, remdesivir, convalescent plasma, dexamethasone and anticoagulation.
- The current consensus is that angiotensin-converting enzyme inhibitors/angiotensin receptor blocker medications should be continued in older patients with increased risk of heart failure or patients with frank heart failure.
- The exact recommendations for anti-coagulation for COVID-19 patients is evolving.

**Outcomes**

- One study determined that 19.7% of COVID-19 positive patients had a cardiac injury.
- Patients with elevated troponin levels and existing cardiovascular disease had higher mortality rates.

## References

[1] Wa Wang D, Hu B, Hu C Clinical characteristics of 138 hospitalized patients with 2019 novel coronavirus–infected pneumonia in Wuhan, China. JAMA 323(11), 1061 (2020). 32031570 10.1001/jama.2020.1585 PMC7042881

[2] Huang C, Wang Y, Li X Clinical features of patients infected with 2019 novel coronavirus in Wuhan, China. Lancet 395(10223), 497–506 (2020). 31986264 10.1016/S0140-6736(20)30183-5 PMC7159299

[3] COVID-19 Map.. https://coronavirus.jhu.edu/map.html.

[4] Hoffmann M, Kleine-Weber H, Schroeder S SARS-CoV-2 cell entry depends on ACE2 and TMPRSS2 and is blocked by a clinically proven protease inhibitor. Cell 181(2), 271–280.e8 (2020). 32142651 10.1016/j.cell.2020.02.052 PMC7102627

[5] Tikellis C, Thomas MC Angiotensin-converting enzyme 2 (ACE2) is a key modulator of the renin angiotensin system in health and disease. Int. J. Peptides 2012, 256294 (2012). 10.1155/2012/256294 PMC3321295 22536270

[6] Inciardi RM, Lupi L, Zaccone G Cardiac involvement in a patient with coronavirus disease 2019 (COVID-19). JAMA Cardiol 5(7), 819 (2020). 32219357 10.1001/jamacardio.2020.1096 PMC7364333

[7] Badawi A, Ryoo SG Prevalence of comorbidities in the Middle East respiratory syndrome coronavirus (MERS-CoV): a systematic review and meta-analysis. Int. J. Infect. Dis. 49, 129–133 (2016). 27352628 10.1016/j.ijid.2016.06.015 PMC7110556

[8] Li B, Yang J, Zhao F Prevalence and impact of cardiovascular metabolic diseases on COVID-19 in China. Clin. Res. Cardiol. 109(5), 531–538 (2020). 32161990 10.1007/s00392-020-01626-9 PMC7087935

[9] Bansal M Cardiovascular disease and COVID-19. Diabetes Metab. Syndr. 14(3), 247–250 (2020). 32247212 10.1016/j.dsx.2020.03.013 PMC7102662

[10] Xiong T-Y, Redwood S, Prendergast B, Chen M Coronaviruses and the cardiovascular system: acute and long-term implications. Eur. Heart J. 41(19), 1798–1800 (2020). 32186331 10.1093/eurheartj/ehaa231 PMC7454513

[11] Oudit GY, Kassiri Z, Jiang C SARS-coronavirus modulation of myocardial ACE2 expression and inflammation in patients with SARS. Eur. J. Clin. Investig. 39(7), 618–625 (2009). 19453650 10.1111/j.1365-2362.2009.02153.x PMC7163766

[12] Shi S, Qin M, Shen B Association of cardiac injury with mortality in hospitalized patients with COVID-19 in Wuhan, China. JAMA Cardiol. 5(7), 802 (2020). 32211816 10.1001/jamacardio.2020.0950 PMC7097841

[13] Clerkin KJ, Fried JA, Raikhelkar J COVID-19 and cardiovascular disease. Circulation 141(20), 1648–1655 (2020). 32200663 10.1161/CIRCULATIONAHA.120.046941

[14] Mishra AK, Lal A, Sahu KK, Sargent J Cardiovascular factors predicting poor outcome in COVID-19 patients. Cardiovasc. Pathol. 49, 107246 (2020). 32640385 10.1016/j.carpath.2020.107246 PMC7263269

[15] Wu J, Stefaniak J, Hafner C Intermittent hypoxia causes inflammation and injury to human adult cardiac myocytes. Anesth. Analg. 122(2), 373–380 (2016). 26505576 10.1213/ANE.0000000000001048

[16] Yang C, Jin Z An acute respiratory infection runs into the most common noncommunicable epidemic – COVID-19 and cardiovascular diseases. JAMA Cardiol. 5(7), 743 (2020). 32211809 10.1001/jamacardio.2020.0934

[17] Epidemiology Working Group for NCIP Epidemic Response, Chinese Center for Disease Control and Prevention. [The epidemiological characteristics of an outbreak of 2019 novel coronavirus diseases (COVID-19) in China]. Zhonghua Liu Xing Bing Xue Za Zhi 41(2), 145–151 (2020). 32064853 10.3760/cma.j.issn.0254-6450.2020.02.003

[18] Arentz M, Yim E, Klaff L Characteristics and outcomes of 21 critically ill patients with COVID-19 in Washington State. JAMA 323(16), 1612 (2020). 32191259 10.1001/jama.2020.4326 PMC7082763

[19] Chen N, Zhou M, Dong X Epidemiological and clinical characteristics of 99 cases of 2019 novel coronavirus pneumonia in Wuhan, China: a descriptive study. Lancet 395(10223), 507–513 (2020). 32007143 10.1016/S0140-6736(20)30211-7 PMC7135076

[20] Sala S, Peretto G, Gramegna M Acute myocarditis presenting as a reverse Tako-Tsubo syndrome in a patient with SARS-CoV-2 respiratory infection. Eur. Heart J. 41(19), 1861–1862 (2020). 32267502 10.1093/eurheartj/ehaa286 PMC7184339

[21] Dong N, Cai J, Zhou Y, Liu J, Li F End-stage heart failure with COVID-19: strong evidence of myocardial injury by 2019-nCoV. JACC. Heart failure 8(6), 515–517 (2020). 32265149 10.1016/j.jchf.2020.04.001 PMC7141452

[22] Kochi AN, Tagliari AP, Forleo GB, Fassini GM, Tondo C Cardiac and arrhythmic complications in patients with COVID-19. J. Cardiovasc. Electrophysiol. 31(5), 1003–1008 (2020). 32270559 10.1111/jce.14479 PMC7262150

[23] ZENGİN S, Avcı S, Yilmaz S Clinical and basic cardiovascular features of patients with COVID-19 admitted to a tertiary care center in Turkey. J. Surg. Med. 4(5), 367–370 (2020).

[24] Guo T, Fan Y, Chen M Cardiovascular implications of fatal outcomes of patients with coronavirus disease 2019 (COVID-19). JAMA Cardiol. 5(7), 811–818 (2020). 32219356 10.1001/jamacardio.2020.1017 PMC7101506

[25] Lippi G, Plebani M Laboratory abnormalities in patients with COVID-2019 infection. Clin. Chem. Lab. Med. 58(7), 1131–1134 (2020). 32119647 10.1515/cclm-2020-0198

[26] Ruan Q, Yang K, Wang W, Jiang L, Song J Clinical predictors of mortality due to COVID-19 based on an analysis of data of 150 patients from Wuhan, China. Intensive Care Med. 46(5), 846–848 (2020). 32125452 10.1007/s00134-020-05991-x PMC7080116

[27] Zeng J-H, Liu Y-X, Yuan J First case of COVID-19 complicated with fulminant myocarditis: a case report and insights. Infection 48(5), 773–777 (2020). 32277408 10.1007/s15010-020-01424-5 PMC7146072

[28] Bangalore S, Sharma A, Slotwiner A ST-segment elevation in patients with Covid-19: a case series. New Engl. J. Med. 382(25), 2478–2480 (2020). 32302081 10.1056/NEJMc2009020 PMC7182015

[29] He J, Wu B, Chen Y Characteristic electrocardiographic manifestations in patients with COVID-19. Can. J. Cardiol. 36(6), 966.e1–966.e4 (2020). 10.1016/j.cjca.2020.03.028 PMC7156155 32299751

[30] Helms J, Tacquard C, Severac F High risk of thrombosis in patients with severe SARS-CoV-2 infection: a multicenter prospective cohort study. Intensive Care Med. 1–10 (2020). 10.1007/s00134-020-06062-x PMC7197634 32367170

[31] Cascella M, Rajnik M, Cuomo A, Dulebohn SC, Di Napoli R Features, evaluation, and treatment of coronavirus (COVID-19). : StatPearls. StatPearls Publishing, Treasure Island (FL) (2020). https://www.ncbi.nlm.nih.gov/books/NBK431128/32150360

[32] Sommerstein Rami, Kochen Michael M, Messerli Franz H, Gräni Christoph Coronavirus disease 2019 (COVID-19): do angiotensin-converting enzyme inhibitors/angiotensin receptor blockers have a biphasic effect? J. Am. Heart Assoc. 9(7), e016509 (2020). 32233753 10.1161/JAHA.120.016509 PMC7428596

[33] “HFSA/ACC/AHA Statement Addresses Concerns Re: Using RAAS Antagonists in COVID-19.”. http%3a%2f%2fwww.acc.org%2flatest-in-cardiology%2farticles%2f2020%2f03%2f17%2f08%2f59%2fhfsa-acc-aha-statement-addresses-concerns-re-using-raas-antagonists-in-covid-19.

[34] Reynolds HR, Adhikari S, Pulgarin C Renin–angiotensin–aldosterone system inhibitors and risk of covid-19. New Engl. J. Med. 382(25), 2441–2448 (2020). 32356628 10.1056/NEJMoa2008975 PMC7206932

[35] Wang M, Cao R, Zhang L Remdesivir and chloroquine effectively inhibit the recently emerged novel coronavirus (2019-nCoV) in vitro. Cell Res. 30(3), 269–271 (2020). 32020029 10.1038/s41422-020-0282-0 PMC7054408

[36] “NIH Clinical Trial Shows Remdesivir Accelerates Recovery from Advanced COVID-19 | NIH: National Institute of Allergy and Infectious Diseases.”. http://www.niaid.nih.gov/news-events/nih-clinical-trial-shows-remdesivir-accelerates-recovery-advanced-covid-19.

[37] Wang Y, Zhang D, Du G Remdesivir in adults with severe COVID-19: a randomised, double-blind, placebo-controlled, multicentre trial. Lancet 395(10236), 1569–1578 (2020). 32423584 10.1016/S0140-6736(20)31022-9 PMC7190303

[38] Yang X, Yu Y, Xu J Clinical course and outcomes of critically ill patients with SARS-CoV-2 pneumonia in Wuhan, China: a single-centered, retrospective, observational study. Lancet Respir. Med. 8(5), 475–481 (2020). 32105632 10.1016/S2213-2600(20)30079-5 PMC7102538

[39] Rajendran K, Krishnasamy N, Rangarajan J, Rathinam J, Natarajan M, Ramachandran A Convalescent plasma transfusion for the treatment of COVID-19: systematic review. J. Med. Virol. 10.1002/jmv.25961 (2020) (Epub ahead of print). 10.1002/jmv.25961 PMC7267113 32356910

[40] “Effect of dexamethasone in hospitalized patients with COVID-19: preliminary report | medRxiv.”. https://www.medrxiv.org/content/10.1101/2020.06.22.20137273v1.

[41] Spyropoulos AC, Levy JH, Ageno W Scientific and standardization committee communication: clinical guidance on the diagnosis, prevention, and treatment of venous thromboembolism in hospitalized patients with COVID-19. J. Thromb. Haemost. 18(8), 1859–1865 (2020). 32459046 10.1111/jth.14929 PMC7283841

[42] Han H, Xie L, Liu R Analysis of heart injury laboratory parameters in 273 COVID-19 patients in one hospital in Wuhan, China. J. Med. Virol. 92(7), 819–823 (2020). 32232979 10.1002/jmv.25809 PMC7228305

[43] Gao L, Jiang D, Wen X-S Prognostic value of NT-proBNP in patients with severe COVID-19. Respir. Res. 21(1), 83 (2020). 32293449 10.1186/s12931-020-01352-w PMC7156898

